# Semantic annotation of consumer health questions

**DOI:** 10.1186/s12859-018-2045-1

**Published:** 2018-02-06

**Authors:** Halil Kilicoglu, Asma Ben Abacha, Yassine Mrabet, Sonya E. Shooshan, Laritza Rodriguez, Kate Masterton, Dina Demner-Fushman

**Affiliations:** Lister Hill National Center for Biomedical Communications, U.S. National Library of Medicine, 8600 Rockville Pike, Bethesda, MD USA

**Keywords:** Consumer health informatics, Question answering, Corpus annotation, Annotation confidence modeling

## Abstract

**Background:**

Consumers increasingly use online resources for their health information needs. While current search engines can address these needs to some extent, they generally do not take into account that most health information needs are complex and can only fully be expressed in natural language. Consumer health question answering (QA) systems aim to fill this gap. A major challenge in developing consumer health QA systems is extracting relevant semantic content from the natural language questions (*question understanding*). To develop effective question understanding tools, question corpora semantically annotated for relevant question elements are needed. In this paper, we present a two-part consumer health question corpus annotated with several semantic categories: named entities, question triggers/types, question frames, and question topic. The first part (*CHQA-email*) consists of relatively long email requests received by the U.S. National Library of Medicine (NLM) customer service, while the second part (*CHQA-web*) consists of shorter questions posed to MedlinePlus search engine as queries. Each question has been annotated by two annotators. The annotation methodology is largely the same between the two parts of the corpus; however, we also explain and justify the differences between them. Additionally, we provide information about corpus characteristics, inter-annotator agreement, and our attempts to measure annotation confidence in the absence of adjudication of annotations.

**Results:**

The resulting corpus consists of 2614 questions (CHQA-email: 1740, CHQA-web: 874). Problems are the most frequent named entities, while treatment and general information questions are the most common question types. Inter-annotator agreement was generally modest: question types and topics yielded highest agreement, while the agreement for more complex frame annotations was lower. Agreement in CHQA-web was consistently higher than that in CHQA-email. Pairwise inter-annotator agreement proved most useful in estimating annotation confidence.

**Conclusions:**

To our knowledge, our corpus is the first focusing on annotation of uncurated consumer health questions. It is currently used to develop machine learning-based methods for question understanding. We make the corpus publicly available to stimulate further research on consumer health QA.

## Background

Consumers increasingly rely on the Internet for their health information needs [1]. A recent Pew Research Center survey found that among the 81% of U.S. adults who use the Internet, 72% have searched online for health information [2]. The same survey revealed that consumers most frequently seek information about specific diseases and treatments (55%) and often self-diagnose using online resources (59%). About half of their searches are concerned with health information needs of family and friends, and most information-seeking activities start with a search engine query (77%).

While search engines have become increasingly sophisticated in retrieving information relevant to search queries, formulating a health information need in a few relevant query terms remains a challenging cognitive task for many consumers [3]. Some research has suggested that short queries used by consumers are not effective in retrieving relevant information [4, 5]. Natural language questions allow the consumers to more fully express their health information needs. Consumers, when they fail to find information using search engines, often turn to online health forums and QA websites, where they can express their health concerns as natural language questions. While such resources are convenient for expressing complex health questions, they also present the additional challenge of *question understanding* for information retrieval systems. Question understanding can broadly be defined as the task of extracting relevant elements or generating a formal representation of a natural language question, which can then be used to formulate a query to a search engine and retrieve relevant answers (*answer retrieval*) [6].

Most research in biomedical QA has focused on clinical questions asked by healthcare professionals [7], which are often succinct and well-formed. In contrast, consumer health questions are rife with misspellings, ungrammatical constructions, and non-canonical forms of referring to medical terms, are fairly long with much background information and multiple sub-questions, and are closer to open-domain language than to medical language [8]. To illustrate, consider the following email received by the customer service of the U.S. National Library of Medicine (NLM):

1. *pls guide us.*
*Dear sir/Madam pls guide us recently we found one of woman staying with us,she coughing and blood coming from mouth so she went to doctor on 2012 they did blood test and sputm test ct scan also they didnt find anything,recently she went to indonesia [LOCATION],they found repot was PROGRESSIVE DISEASE,ACTIVE LUNG TBINTHE RIGHT B2 AND B4 SEGMENS,THE EXUDATIVE LESIONS IS INCREASING WITH SMALL CAVITY.so what we have to do for her is this contages,who is the pople staying with her need to do test ?pls guide me thank u my contact [CONTACT]*

Three sub-questions can be distinguished: one regarding treatment options for lung tuberculosis, another asking whether this disease is contagious and the third asking how to get tested. The question includes background information that can be considered irrelevant for answer retrieval (that the patient saw a doctor in 2012 and travelled to Indonesia). It also contains spelling and grammar errors that would render it difficult to process with standard NLP tools. For example, the disease in question has a word break error and is abbreviated and *contagious* is misspelled, making both sub-questions very difficult to parse automatically. Based on such characteristics, it has been suggested that consumer health QA systems should be designed with considerations different from those underlying QA systems targeting healthcare professionals [8].

NLM receives health-related questions and queries from a wide range of consumers worldwide. We have been developing a consumer health QA system to assist various NLM services in answering such questions. Question understanding is a core component of this system. As is typical with other NLP tasks, annotated corpora are needed to develop and evaluate automated tools addressing this task. Our earlier work relied on a small corpus [9], which was sufficient for evaluating the narrow focus of the prototype system developed, but was not large enough to train QA systems. In other work, we relied on curated consumer health questions [10, 11]. In the present study, we aimed to address the gap by annotating a corpus of unedited, uncurated consumer health questions. We annotated 2614 consumer health questions with several semantic layers: named entities, question topic, question triggers, and question frames. The majority of the questions (67%) come from consumer health emails received by the the U.S. National Library of Medicine (NLM) customer service and are relatively long questions (Example 1). We refer to this subset as *CHQA-email*. The rest of the questions (33%) have been harvested from the search query logs of MedlinePlus, a consumer-oriented NLM website for health information, and are generally much shorter (*CHQA-web*). Despite being posed as search queries, these questions are expressed as natural language questions, rather than keywords. An example question from CHQA-web is provided below:

(2) *what teeth are more likely to get cavities?*

In a previous study, we annotated a subset of the questions in CHQA-email with named entities [12]. The present study builds upon and significantly extends that work. Here, we describe corpus characteristics, annotation schemes used, annotation steps taken, and statistics on the resulting dataset. Additionally, we report a small study to automatically assess confidence of annotations, when annotation adjudication can be impractical. We make the resulting annotated corpus publicly available at https://bionlp.nlm.nih.gov/CHIQAcollections/CHQA-Corpus-1.0.zip. To our knowledge, the corpus is unique for its focus on uncurated consumer health questions and the level and breadth of semantic detail it incorporates. We believe that, with its size and coverage, it can stimulate further research in consumer health QA.

## Related work

Corpus construction and annotation has been critical for the progress made in NLP in the past several decades [13–15]. Biomedical NLP has also benefitted significantly from numerous domain-specific corpora annotated for linguistic and semantic information. Most annotation efforts have focused on biomedical literature and clinical reports and a small number on other text types such as drug labels and social media text. The types of information annotated have ranged from syntactic structure [16, 17] to semantic phenomena, including named entities/concepts [18–22], relations/events [19, 23–25], assertion/uncertainty/negation [19, 26], coreference [27–29], and rhetorical structure [30, 31]. Community-wide shared task competitions have relied on such corpora to advance the start-of-the-art in biomedical NLP [19, 32–35].

Most biomedical QA research has focused on information needs of healthcare professionals, and several corpora have been used for this purpose. The Clinical Questions Collection, maintained by the NLM, is a repository of 4654 questions collected by Ely et al. [36] and D’Alessandro et al. [37] at the point of care. Original questions, their short and general forms, their topics (e.g., *management*, *diagnosis*, *epidemiology*), keywords (e.g., *Polymenorrhea*, *Menstruation Disorders*) as well as physician and patient information are provided. This collection has been used to develop several machine learning-based QA systems, such as AskHERMES [38] and MiPACQ [39]. Other smaller corpora also exist; for example, Parkhurst Exchange and Journal of Family Practice^1^ maintain curated question sets that have been used to develop CQA, a clinical QA system based on evidence-based medicine principles [40]. Patrick and Li [41] presented a set of 446 clinical questions from staff in an intensive care unit asking for patient-specific information from their electronic health records and created a question taxonomy based on answering strategies. Text REtrieval Conference (TREC) Genomics track provided a platform to study QA for biological research [42]. A set of 36 questions asking for lists of specific entities (antibodies, pathways, mutations, etc.) was manually developed to represent the information needs of the biologists (e.g., *What [MUTATIONS] in the Raf gene are associated with cancer?*). More recently, BioASQ challenge also focused on biomedical QA and presented a corpus of 311 questions, categorized by the type of answers they require: yes/no, factoid, list, and summary [43].

Two approaches to modeling and formally representing biomedical questions have been distinguished: empirical and domain-based models [6]. In the empirical approaches, the questions are categorized into a limited number of generic question types [36, 41, 44]. Domain-based models provide a high-level definition of the main entities and actors in the domain and their relationships. For example, the PICO framework represents the elements of a clinical scenario with four elements, population/problem (P), intervention (I), comparison (C), outcome (O), and has been used as the basis of the CQA system [40]. Other formal approaches have also been used to represent clinical questions; for example, they have been represented as concept-relation-concept triples [45, 46], Description Logic expressions [47], SPARQL queries [48], and *λ*-calculus expressions [49].

Early research on consumer health information seeking focused on analysis of consumer health queries [4, 50]. Based on the finding that such queries are not effective in finding relevant information [4, 5], several other small-scale studies specifically analyzed consumer health questions [3, 51–53]. For example, Zhang [3] analyzed 276 health-related questions submitted to Yahoo! Answers, a community QA website, on several dimensions, including linguistic style and motivation, and found that these questions primarily described diseases and symptoms (accompanied by some patient information), were fairly long, dense (incorporating more than one question), and contained many abbreviations and misspellings. Slaughter et al. [52] manually annotated the semantics of a small number of consumer health questions and physician answers using Unified Medical Language System (UMLS) Semantic Network and found that both patients and physicians most commonly expressed causal relationships. More recently, Roberts and Demner-Fushman [8] studied how consumer questions differed from those asked by professionals and analyzed 10 online corpora (5 consumer, 5 professional, including some that have been discussed above) at lexical, syntactic, and semantic levels using a variety of NLP techniques. Their findings largely mirror those of Zhang [3]. Additionally, they found substantial differences between consumer corpora and showed that consumer health questions are closer to open-domain language than to medical language. Based on these findings, they suggest that consumer health QA systems need to be designed with a different set of considerations, instead of naively adapting a professional QA system. Liu et al. [54] developed a SVM classifier to distinguish consumer health questions from professional questions, and used questions from Yahoo! Answers and the Clinical Questions Collection for training.

We developed several consumer health corpora in the context of our consumer health QA project. In early work, we used a small set of 83 consumer health questions about diseases/conditions (mostly genetic), which consisted of relatively simple questions received by the NLM customer service and frequently asked questions collected from Genetic and Rare Disease Information Center (GARD)^2^, to develop a rule-based approach to extract question type and question theme [9]. GARD also formed the basis of annotation of 13 question types on 1,467 questions focusing on genetic diseases [10]. These question types include *anatomy* (location of a disease), *cause* (etiology of a disease), *information* (general information about a disease), and *management* (treatment, management, and prevention of a disease), among others. The average inter-annotator agreement (Cohen’s *κ*) for question types was found to be 0.79 (substantial agreement). Recognizing that consumer health questions are complex with multiple sub-questions related by a central theme (focus), Roberts et al. [11] also annotated the same GARD dataset with question focus and question decomposition, where individual sub-questions are identified within complex questions, and each sub-question is annotated for several elements, including the central theme (*focus*), contextual information (*background*) and the actual question, at sentence and phrase levels. *Background* sentences were further classified into one of several medical categories, such as diagnosis, symptoms, or family history. The average inter-annotator agreement was found to be 0.91 for *focus* and 0.72 for *background* class. These GARD-based datasets have been used to train and evaluate question type classifiers [55] as well as focus recognizers and question decomposition classifiers [56, 57]. The questions in the GARD dataset, even though they were asked by consumers, are curated; thus, they are well-formed with few misspellings, and represent an intermediate step between simple questions addressed in [9] and the more typical, complex consumer health questions. Consequently, while good classification performance is reported on the GARD dataset, performance drops significantly on the more complex questions received by NLM [57]. To address this gap, in recent years, we focused on annotation of uncurated consumer health questions received by NLM, without any simplifying assumptions. Considering that misspellings are a major obstacle in question understanding and that off-the-shelf spelling correction tools do not work well on customer health questions, we developed a spelling correction dataset that consists of 472 NLM questions [58]. In this dataset, we annotated non-word, real word, punctuation, as well as word break errors. We also developed an ensemble method that uses edit distance, corpus frequency counts, and contextual similarity to correct misspellings. Finally, we created a dataset of 1548 questions taken from the same resource, de-identified them for personal health information, and annotated them for named entities (CHQ-NER) [12]. Each question was double-annotated using 16 named entity types (e.g., ANATOMY, DRUG_SUPPLEMENT, PROBLEM, PROCEDURE_DEVICE, PERSON_ORGANIZATION), determined based on a round of practice annotation. The annotation was performed in both manual and assisted mode, in which existing named entity recognition tools were used to pre-annotate questions. After double-annotation of questions, they were reconciled by one of the expert annotators.

Moderate agreement (0.71 F_1_ agreement with exact matching) was achieved, with slightly higher agreement in assisted mode (0.72).

## Methods

In this section, we first describe our approach to representing consumer health questions. Next, we discuss corpus construction and the semantic annotation that we performed on this corpus in depth. We conclude this section by providing details on our attempt to automatically estimate confidence scores of semantic annotations, when full adjudication is not feasible due to personnel/time constraints.

### Question representation

We briefly discussed several approaches to biomedical question representation above: empirical, domain-based, and formal. Our approach to representing consumer health questions can be viewed as a hybrid method. Like empirical approaches, we begin with existing questions and categorize them into a limited number of generic question templates, and, like formal approaches, we create structured representations of the information in these generic questions that we call *question frames*. Frame representation is similar to a predicate-argument structure, a semantic representation in which a predicate is linked to its arguments and the semantic roles of the arguments, such as THEME and AGENT, are specified [59]. A question frame consists of a question trigger indicating the question type (similar to predicate), one or more THEME arguments, and a set of optional arguments with other semantic roles, all linked to their textual mentions. All arguments of a frame correspond to named entities. Each sub-question in a consumer health question is represented with a separate frame. To illustrate, consider the question we discussed earlier, reproduced in Example 3. An example representation of this question consists of the frames in Table 1.

(3) *pls guide us.*

**Table 1:** Frame representation of the question in Example 3

Frame 1	
Question type	*do*:treatment
Theme	*ACTIVE LUNG TB*: problem
Frame 2	
Question type	*contages*:susceptibility
Theme	*ACTIVE LUNG TB*: problem
Frame 3	
Question type	*test*:diagnosis
Theme	*ACTIVE LUNG TB*: problem

The question is represented with three frames, each composed of two elements, question type and theme. The content of each element is shown as a mention:TYPE pair
*Dear sir/Madam pls guide us recently we found one of woman staying with us,she coughing and blood coming from mouth so she went to doctor on 2012 they did blood test and sputm test ct scan also they didnt find anything,recently she went to indonesia [LOCATION],they found repot was PROGRESSIVE DISEASE,ACTIVE LUNG TBINTHE RIGHT B2 AND B4 SEGMENS,THE EXUDATIVE LESIONS IS INCREASING WITH SMALL CAVITY.so what we have to do for her is this contages,who is the pople staying with her need to do test ?pls guide me thank u my contact [CONTACT]*

The first frame indicates that the information request contains a question about the treatment of active lung tuberculosis, triggered by the verb *do* in *what we have to do for her*. Note that while a THEME argument is required for each frame, we only specify other arguments if they are likely to be relevant for answer retrieval. For example, in the first frame, one could specify *woman* as an argument with PATIENT role (i.e., the person to undergo treatment); however, since gender is unlikely to be significant in answering this particular question, its explicit representation is not required. In this sense, our representation is driven more by pragmatic concerns for question answering than by completeness.

**Fig. 1:**
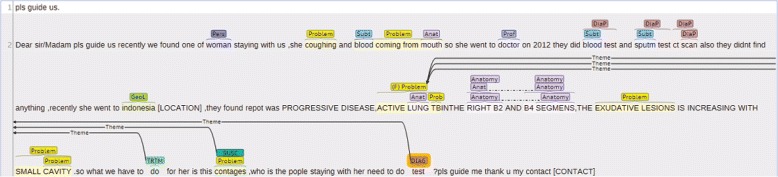
*Brat* annotation for the consumer health question in Example 3. Named entities and question triggers are indicated with text spans and the question frames are represented as edges between the question trigger and named entities that act as arguments. Question topic (*ACTIVE LUNG TB*) is indicated with (F) next to its named entity category. Named entity categories are sometimes abbreviated: ANATOMY (Anat), DIAGNOSTIC_PROCEDURE (DiaP), GEOGRAPHIC_LOCATION (GeoL), PERSON_POPULATION (Pers), PROFESSION (Prof), SUBSTANCE (Subt). For question type categories, the abbreviated forms are: DIAGNOSIS (DIAG), SUSCEPTIBILITY (SUSC), and TREATMENT (TRTM)

Our prior research suggested that when the question topic^3^ (often the THEME of a sub-question) and the question types in an information request are known, authoritative answers can be found in 60% of the cases [60]. Taking this into account, we also explicitly indicate the question topic in question representation. The question topic is indicated as a named entity attribute. In the question above, the named entity corresponding to the mention *ACTIVE LUNG TB* is marked as the question topic. The annotation of this question is illustrated in Fig 1.

### Corpus construction and annotation

Our corpus consists of two parts: a set of 1740 questions harvested from consumer health emails received by the NLM customer service between 2013 and 2015 (CHQA-email) and another set of 874 questions harvested from MedlinePlus search query logs (CHQA-web).

#### CHQA-email

CHQA-email is an extension of an earlier corpus (CHQ-NER), reported in [12], and supersedes it. That corpus consisted of 1548 consumer health information requests received by the NLM customer service in 2014-15 and manually labeled as disease and drug questions by the staff. These requests were annotated for named entities. For details of the question selection procedure, see [12]. Protected health information (PHI), such as names, locations, contact information, has been manually replaced with surrogates in these questions.

As part of the present study, we extended CHQ-NER with 195 additional questions. Furthermore, 2 duplicate questions and 1 non-question request from the original corpus were dropped, bringing the total number of questions to 1740. One hundred fourty six of the new questions come from the set of 300 questions that were considered in a study that investigated whether authoritative answers to consumer health questions can be found in MedlinePlus and other online resources [60]. In that study, question types and topics were marked. Fourty nine questions came from a set which we used for an earlier, experimental annotation of question type, focus, and frames (unpublished). We incorporated existing annotations in these sets as pre-annotations to be used in subsequent steps. Both these additional sets of questions were then de-identified and annotated with named entities, following the guidelines for CHQ-NER [12]. One expert annotator (SES) performed the annotation, she and another expert (DDF) then discussed and adjudicated these named entity annotations. At this stage, all 1740 questions had been fully annotated for named entities.

The rest of annotation of this part of the corpus proceeded as follows: first, all annotators annotated 20 questions for the elements under consideration (question topics, triggers/types, and frames). As the question type categories, we used those that were used in annotating GARD questions [10]. As this step was exploratory and included drug questions which we had not considered before, we allowed the annotators to propose additional question types. Six annotators were involved in this practice step. After a round of discussion and adjudication, we extended the question type categorization, defined three semantic roles (THEME, KEYWORD, and EXCLUDE_KEYWORD), and prepared annotation guidelines. We did not calculate inter-annotator agreement for these practice questions and only make available the final, adjudicated annotations.

Next, we sliced the rest of corpus (1720 questions) such that each annotator was allocated roughly the same number of questions, and each shared with all the other annotators approximately the same number of questions. Seven annotators were involved, although the additional annotator (Ann7) annotated significantly fewer than the others due to scheduling conflicts. The questions initially allocated to her but could not be annotated were redistributed among four annotators in such a way to ensure that each question was still double-annotated and the questions were fairly distributed. The resulting number of questions annotated by each annotator is given in Table 2. At this step, the annotators were allowed to modify the original named entity annotations if they deemed it necessary for topic/frame annotation. After this step was completed, we automatically checked the annotations for consistency, which involved addressing two common problems observed in annotation: first, we ensured that each question contained a question topic annotation and second, we checked that each frame contained a single THEME argument, except when the question type is one that allows multiple THEME arguments, such as COMPARISON.

**Table 2:** The number of questions annotated by each annotator in CHQA-email

Annotator	# questions
Ann1	565
Ann2	495
Ann3	553
Ann4	489
Ann5	554
Ann6	544
Ann7	240

We calculated inter-annotator agreement for question triggers/types, frames with and without optional elements, and question topic. Inter-annotator agreement is calculated as the micro-average F_1_ score when one set of annotations is considered the gold standard [61].

Considering the length of questions, the average number of annotations per question, and the number of annotators involved, adjudicating this set is a daunting task^4^. Therefore, instead of adjudication, we chose to investigate whether confidence scores can be automatically estimated for annotations. More details about this process are given at the end of this section.

#### CHQA-web

In contrast to CHQA-email, the second part of the corpus, CHQA-web, has been fully annotated from scratch in this study. MedlinePlus provides search capabilities that are geared towards traditional keyword searches; however, a significant number of searches are posed as natural language questions. Such searches often fail. To investigate whether we can address such questions automatically and to create a counterpoint to customer service information requests, we harvested a set of short questions from MedlinePlus search query logs. These questions were asked in the period from May 2015 to April 2016. We looked for *wh*-words to identify these questions in the query logs: *how, what, when, where, which, who, why*. We also included queries ending with question marks and those that start with an auxiliary (*is it, can, could, do, does*). For each category, we selected 70 most frequent questions as well as 70 random questions. After removing duplicates and near-duplicates using string matching heuristics, we were left with 1180 questions. In contrast to CHQA-email, we did not assess whether these were all legitimate questions or were answerable; instead, we used two labels, NOT_ANSWERABLE and NOT_QUESTION, during the annotation process to mark such questions. Recognizing that our heuristics for removing duplicates may not be adequate, we used another label, DUPLICATE, to indicate such cases.

In a practice step, the same six annotators annotated and adjudicated 100 of these questions using the guidelines for CHQA-email. During this process, the guidelines were updated: some question types were consolidated, a couple of new types were added, and the types of semantic roles were expanded.

Next, four of the annotators double-annotated the rest of the questions (n=1,080). Instead of pairing all annotators, we only paired those with clinical expertise (n=2) with those without (n=2), leading to four annotator combinations. In the end, each annotator labeled 540 questions. Since these questions were relatively short and there were fewer annotator pairs, we chose to adjudicate these annotations. Each pair adjudicated the questions that they both annotated. As in CHQA-email, we calculated inter-annotator agreement using micro-average F_1_ score. Fourty eight questions were adjudicated as NOT_QUESTION, 203 were deemed NOT_ANSWERABLE and 55 were found to be DUPLICATE, bringing the total number of annotated questions in this part down to 874.

#### Annotation

Seven annotators participated in various stages of the annotation, depending on their availability. All annotators had experience in working with biomedical text. Two annotators (LR, DDF) have clinical expertise, two are trained as medical librarians (SES, KM), and four have experience in natural language processing (HK, ABA, YM, and DDF). We used *brat* annotation tool [62] for all stages of annotation. Named entities and question triggers were annotated as terms, and frames were annotated as events in *brat*. Question topic was specified as an attribute of the relevant named entity.

In the rest of this subsection, we provide details about annotation of these semantic layers and highlight the differences in their annotation between the two parts of the corpus.

##### Named entity annotation

Named entity annotation in CHQA-email essentially follows the principles of CHQ-NER [12].

In CHQ-NER, we annotated 16 broad categories of named entities (ANATOMY, CELLULAR_ENTITY, DIAGNOSTIC_PROCEDURE, DRUG_SUPPLEMENT, FOOD, GENE_PROTEIN, GEOGRAPHIC_LOCATION, LIFESTYLE, MEASUREMENT, ORGANIZATION, PERSON_POPULATION, PROBLEM, PROCEDURE_DEVICE, PROFESSION, SUBSTANCE, and OTHER). Nested entities and multi-part, non-contiguous annotations were allowed. Annotation was performed both manually and with assistance from several named entity recognizers [63–65].

For the present study, recognizing that many of the entities with OTHER type corresponded to physiological functions (e.g. *pregnancy*), we added a new named entity type ORGANISM_FUNCTION. We use the UMLS [66] definition for Organism Function semantic type for this new category: “A physiologic function of the organism as a whole, of multiple organ systems, or of multiple organs or tissues”. To ensure that annotations are consistent, one of the annotators (DDF) examined all strings annotated as OTHER in CHQ-NER and identified those that can be considered organism functions. We then automatically relabeled these mentions, originally labeled OTHER, as ORGANISM_FUNCTION in CHQA-email.

For CHQA-web, we considered 18 categories: 16 categories above in addition to ORGANISM_FUNCTION and RESEARCH_CUE. The latter, though not common, is a category that was devised to indicate that the consumer is interested in information that goes beyond typical consumer health information.

Example RESEARCH_CUE mentions include *new* and *latest information*. In contrast to CHQA-email, we did not consider nested entities for CHQA-web, as the annotators generally found the annotation of nested entities challenging, although such entities can provide a more precise evaluation for named entity recognizers, as illustrated in [12].

The full list of named entity categories used in annotation, with brief definitions, examples, and relevant UMLS semantic types, is provided in Table 3.

**Table 3:** Named entity categories with definitions, examples, and relevant UMLS semantic types

Entity type	Brief definition	Examples	UMLS semantic types
*Named entity categories from* [12]			
anatomy	Includes organs, body parts, and	*head*, *neck*, *gum*	Body System,
	tissues.		Anatomical Structure
cellular_entity	Includes anatomical entities at the	*hemoglobin*,	Cell,
	molecular or cellular level.	*giant cell*	Cell Component
diagnostic_	Includes tests and procedures used	*biopsy*, *hemoglobin*,	Diagnostic Procedure,
procedure	for diagnosis	*iron levels*	Laboratory Procedure
drug_supplement	Includes substances used for	*atenolol*,	Clinical Drug,
	therapeutic purposes.	*atenolol 50 mg*,	Vitamin
		*campho-phenique*	
food	Refers to specific nutritional	*eggs*, *breads*, *meat*	Food
	substances.		
gene_protein	Includes specific genes and gene	*BRCA1*,	Gene or Genome,
	products.	*BRCA1 gene*,	Enzyme
		*GLUT4 protein*	
geographic_	Includes countries, cities, etc.	*India*, *Singapore*	Geographic Area
location			
lifestyle	Refers to daily and recreational	*smoking*, *yoga*	Daily or Recreational
	activities.		Activity
measurement	A quantity that is a core attribute of a named entity, such as dosage of a drug.	*10mg*, *2%*	Quantitative Concept
organization	Includes institutions as well as their	*navy*, *California*	Organization
	subparts.	*Hospitals*	
person_	Includes individuals (gender, age	*daughter*, *female*,	Age Group,
population	group, etc.) and population groups.	*war veteran*,	Population Group
		*16 year old*	
problem	Includes disorders, symptoms,	*HIV*, *cholesterol*,	Disease or Syndrome,
	abnormalities, and complications.	*broke*,	Neoplastic Process
		*autoimmune disease*	
procedure_device	Refers to procedures or medical	*shingles treatment*,	Medical Device,
	devices used for therapeutic	*nephrolithotomy*,	Therapeutic or
	purposes as well as unspecific	*implants*	Preventive Procedure
	interventions.		
profession	Includes occupations, disciplines, or	*dermatologist*, *dr*,	Professional or
	areas of expertise.	*surgeon*	Occupational Group
substance	Includes chemicals, hazardous	*iron*, *cholesterol*,	Inorganic Chemical,
	substances, and body substances.	*blood*, *alcohol*	Biologically Active
			Substance
other	Includes entities that are relevant to	*more than once*	Temporal Concept
	question understanding, but do not		
	fit in one of the categories above.		
*Named entity categories added in the present study*			
organism_function	Refers to physiological functions of the organism.	*pregnancy*	Organism Function
research_cue	Indicates that consumer is interested in research information.	*new*, *latest information*	Qualitative Concept

##### Question topic annotation

Question topic is the central theme (a disease, a drug, etc.) of a question. Each legitimate question is expected to have at least one topic, but multiple topic elements can be annotated, especially in questions about relationships between two diseases or drugs. In the earlier example, *ACTIVE LUNG TB* is the question topic, while there are two topics in the following question in bold:

(4) *I’d like to learn more about* ***megalocytic interstitial nephritis*** *with* ***malakoplakia***.

All mentions of the topic term, including synonyms and misspellings, were expected to be annotated. For example, in the following question, synonyms *anosmia* and *loss of smell* are both legitimate topics.

(5) ***ANOSMIA***.
*This site tells me that ongoing research may solve my problem some day. Please, could I be informed if such result does happen? I live alone, aged 78, and this complete* ***loss of smell*** *has been my great problem for quite a while.*

When considering nested entities for question topics, annotators were instructed to label the term as topic at the nesting level that is appropriate for the question. For example, in the following question, both mentions of *ropinirole HCl* as well as *repinirole HCl 0.25 mg* and *ropinirole HCl 0.5 mg* were annotated as named entities. With this instruction, annotators were expected to label mentions of *ropinirole HCl* as the topic, since the dosage/form of this drug is not relevant to the primary question.

(6) *Ropinirole HCl 0.25 and ropinirole HCl 0.5 mg. Please tell me if these meds are gluten-free.*

These principles applied to question topic annotation in CHQA-email. For short questions in CHQA-web, based on the practice annotation, we chose not to explicitly annotate question topic, since this almost always corresponded to the THEME argument of the question frame.

##### Question type annotation

We based our practice annotation of CHQA-email on the 14 question type categories proposed for disease questions in [10]. Discussion/adjudication of practice questions led to an expanded set of 33 categories. These 33 categories can be analyzed in three distinct groups.

- *General question types:* These categories can apply to any type of entity (comparison, information, and other_question).
- *Problem question types:* These categories apply to diseases/conditions, and other problems (e.g., cause, complication, diagnosis, inheritance, prognosis, and treatment). A subset of these categories also apply to entities of procedure and medical device types, since problems can arise from their usage (e.g., complication, prognosis, and support_group).
- *Intervention question types:* These categories apply to drugs, supplements, as well as procedures and medical devices (e.g., action, indication, side_effect, and usage).

We provide some further details in Table 4. The last column indicates whether the question type was among the 14 categories proposed by Roberts et al. [10].

**Table 4:** Question type categories in CHQA-email with their definitions and some commonly used triggers

Question type	Brief definition	Example triggers	In [10]
*General question types*			
comparison	Concerned with comparison of several entities (often of the same type)	*comparison*, *differences*	
information	General information about an entity	*information*, *types*	✓
other_question	Information not covered with other types	*prepare*, *cover*	✓
*Problem question types*			
cause	Cause of a disease	*cause*, *trigger*	✓
complication*	Longer term effects of a disease	*risk*, *damage*	✓
diagnosis	Methods of diagnosing a disease	*diagnose*, *detection*	✓
effect*	Unspecific effects of a disease	*affect*, *link*, *related*	✓(a)
frequency	Prevalence of a disease	*statistics*, *prevalence*	✓(b)
inheritance	Inheritance patterns of a disease	*passed*, *genetic*	✓(b)
lifestyle_diet*	Lifestyle/diet changes after a disease	*okay*, *precautions*	
location*	Body location of a disease	*areas*, *occur*	✓(c)
person_organization*	Individuals/organizations specializing in a disease	*find*, *consult*	✓(d)
prevention	Methods of prevention for a disease	*prevent*, *save from*	✓(e)
prognosis*	Life expectancy and quality of life for a disease	*recovery*, *how long*	✓
support_group*	Support groups for a disease	*support groups*, *recommend*	✓(d)
susceptibility	Transmission of a disease	*transmitted*, *spread*	✓
symptom	Signs and symptoms of a disease	*symptom*, *normal*	✓(f)
treatment	Treatment, cure, management of a disease	*cure*, *help*, *improve*	✓(e)
*Intervention question types*			
action	How a drug acts in the body	*start working*	
alternative	Alternatives for an intervention	*alternative*, *replacement*	
contraindication	Conditions in which an intervention should be avoided	*take if*, *hurt*	
cost	Pricing of an intervention	*cost*, *rate*	
dosage	Appropriate dosage of a drug	*dosage*, *administration*	
indication	Conditions to use an intervention	*for*, *given to*	
ingredient	Ingredients of a drug	*in*, *made from*	
interaction	Interactions between drugs	*reaction*, *safe to take*	
long_term_effect	Long term consequences of an intervention	*cause*, *long term effect*	
overdose	Consequences of a substance overdose	*do*, *hurt*	
side_effect	Short-term, adverse reactions to an intervention	*side effect*, *poisonous*	
storage_disposal	Instructions for storing/disposing a drug	*expire*, *stability*	
tapering	Instructions for how to stop using a drug	*weaning*, *withdrawal*	
usage	Patient instructions for an intervention	*applying*, *take*	
drug_question	Other intervention question (e.g., drug form)	*come in*, *potent*	

Notes: (*) Applies to procedures/medical devices, as well. (a) As other_effect. (b) As susceptibility. (c) As anatomy. (d) As person_org. (e) As management. (f) As manifestation

For question type annotation in CHQA-web, this scheme was modified in several ways, resulting in a somewhat simplified categorization that consists of 26 classes. First, some question types that do not occur frequently were merged into other categories that can be considered their supertypes. For example, in CHQA-email annotation, we split the question type SUSCEPTIBILITY as defined in Roberts et al. [10] into three types that address different but related characteristics of diseases: FREQUENCY, INHERITANCE, and SUSCEPTIBILITY. Considering that answers to such questions are likely to be found in the same passages, for annotation convenience and expediency, we merged these types back into SUSCEPTIBILITY in CHQA-web. The consolidated question types are the following:

- {complication, long_term_effect, overdose, side_effect} →complication
- {person_organization, support_group} →person_organization
- {frequency, inheritance, susceptibility} →susceptibility
- {dosage, storage_disposal, tapering, usage} →usage

We generalized the question type EFFECT to indicate underspecified relations between two or more named entities, and changed its name to ASSOCIATION for clarity. Finally, we added two new question types. DIAGNOSE_ME identifies questions asking for diagnosis from a set of symptoms. For example, this question type was annotated for the following question (the trigger is in bold).

(7) *my waist turn red like i have hives but it’s not and its not achy or itchy,* ***what*** *do u think* ***it is***?

While we specifically avoided annotating such questions in CHQA-email, since they were considered beyond the scope of a QA system provided by a medical library, we added such annotations in CHQA-web to allow further experiments. The other question type, TIME, identifies questions asking for time/duration of an event. An example is given below.

(8) *who gets vaccines and* ***when***?

The annotators were instructed to annotate the minimal trigger expression, except when two equally valid triggers are conjoined. In the first question in Example 1, the question trigger is *take*, rather than *can take* or *if I can take*. In the second question, since both *reduce* and *remove* can trigger the same question type, the question trigger is taken as *reduce or remove*.

(9)
*I would like to ask if I can* ***take*** *glutathione supplement.*
*fibroadenomas.…taking medicine is help to* ***reduce or remove*** *this?…*

##### Question frame annotation

In CHQA-email, question frames were annotated using the 17 named entity categories and 33 question types discussed above. A question frame in this context minimally consists of two elements: a trigger indicating the question type and a THEME argument, which corresponds to a named entity. The type of the question frame is inherited from the trigger. Most questions types allow a single THEME argument, with the exceptions of EFFECT, which allows multiple THEME arguments, and COMPARISON, which requires multiple THEME arguments. Restrictions on the type of named entities that can fill the THEME role are specified in Table 5.

**Table 5:** Restrictions on THEME arguments in CHQA-email

Question types	Theme restrictions
*General question types*	
comparison	entity{2,}
information, other_question	entity
*Problem question types*	
cause, diagnosis, frequency, inheritance, prevention, susceptibility, symptom, treatment	problem
complication	problem, procedure_device, lifestyle, food
effect	problem+, procedure_device+
lifestyle_diet, location, prognosis	problem, procedure_device
person_organization, support_group	problem, procedure_device, diagnostic_procedure
*Intervention question types*	
action, dosage, drug_question, ingredient, overdose	drug_supplement, substance
alternative, cost, indication, long_term_effect, side_effect, usage	drug_supplement, substance,procedure_device, diagnostic_procedure
contraindication, storage_disposal, tapering	drug_supplement, substance, procedure_device
interaction	drug_supplement{2,}, substance{2,}

{2,} indicates cardinality of at least two, + indicates at least one argument

In CHQA-web, question frame annotation involved 26 question types and 18 named entity categories. We lifted the type restrictions for theme arguments for this part of the corpus, because the annotators found them too restrictive. However, the cardinality restrictions remained in place. For example, INTERACTION still required at least two THEME arguments. Two additional question types, DIAGNOSE_ME and TIME, were allowed multiple themes and a single theme, respectively.

Two parts of the corpus also differ with respect to non-THEME semantic roles. In CHQA-email, two generic semantic roles were defined: KEYWORD, and EXCLUDE_KEYWORD. KEYWORD arguments indicate named entities that are expected to be helpful in finding more specific answers to a question. EXCLUDE_KEYWORD arguments are those that should be used to exclude some potential answers. While KEYWORD can be viewed as a generic semantic role and can correspond to more typical semantic roles, such as AGENT, PATIENT, PURPOSE, EXCLUDE_KEYWORD is unique and is meaningful in a question answering context. Examples in Table 6 illustrate these roles.

**Table 6:** Illustration of KEYWORD and EXCLUDE_KEYWORD semantic roles in question frames

Question	*Is it ok to drink quinine in seltzer water to ease leg cramps?*	
Frame	Question type	*ease*:treatment
	Theme	*leg cramps*: problem
	Keyword	*quinine*: substance
	Keyword	*seltzer water*: substance
Question	*My father is 70 years old, his arteries in the both limbs have blockage.*	
	*Doctor’s suggest to go for amputation but there is high risk in it. Please*	
	*suggest treatment without amputation.*	
Frame	Question type	*treatment*:treatment
	Theme	*arteries in the both limbs have blockage*: problem
	ExcludeKeyword	*amputation*: procedure_device

The contents of frame elements are shown as *mention*:TYPE pairs

In CHQA-web, we used fine-grained roles, more in line with typical roles used in semantic role labeling. On the other hand, EXCLUDE_KEYWORD was not considered. The fine-grained roles used are as follows:

- agent indicates the entity that performs the action involving the theme. In the clinical domain, inanimate objects, such as drugs, procedures and devices could be agents. For example, in the first example in Table 6, both keyword arguments can be annotated as agents.
- locative indicates the location where the action involving the theme occurs (primarily, the body parts). *leg* in the question *parkinson’s disease best drugs for legs?* can be annotated with this role in a treatment frame.
- purpose indicates the reason/goal for which the action is performed. *eczema* in the question *how many mg. of zinc daily for eczema patients?* can be annotated with this role in a usage frame.
- patient indicates the experiencer of the action. *baby* in the question *what if a mother quits intravenous drug use at five months what side effect will baby have?* (a complication frame).
- restrictor indicates the context in which the action occurs or a restriction on the type of information being sought. *doctor* in the question *what kind of doctor would i see for calcification?* (a person_organization frame).
- temporal indicates the time at which the action occurs or the duration of the action. *4 years ago* in the question *is zoster vaccine effective if one had shingles 4 years ago?* (an indication frame)
- research indicates the role of research_cue entities. *literature* in the question *review of literature of cancer stress* (an information frame).

A question frame can contain multiple arguments with such optional roles. We did not stipulate any semantic restrictions on the named entities that can fill these roles, even though for example, the PATIENT role is likely to be filled with an entity of PERSON_POPULATION type, and the RESEARCH role should be filled with a RESEARCH_CUE entity.

In CHQA-email, we associated a frame with the attribute RESEARCH to indicate that the person asking the question is interested in information that goes beyond consumer health information. In CHQA-web, as noted above, we specifically annotated terms indicating such information need as named entities (RESEARCH_CUE), in order to allow training of machine learning models that automatically learn such phrases.

When there are multiple mentions of a term that fills a specific role, the annotators were instructed to use the mention that is syntactically linked to the question type trigger, if possible. Otherwise, they select the closest mention that precedes the trigger. Similarly, if there are multiple potential triggers, annotators were expected to select the trigger that is syntactically related to the theme or is the trigger closest to it.

The same question is sometimes asked multiple times in the same information request, especially in long requests. Annotators were expected to annotate all instances of such questions. We also found that some questions can be represented from multiple perspectives. Consider the example below:

(10) *questions about the effect of L-Leucine.*
*Hello,do you have any introduction about L-Leucine on the effect of treat cancer?If so,could you kindly tell me details?*

This can be formalized in one of two ways:

- A treatment frame where *cancer* fills the theme role and *l-leucine* is an agent (in essence, corresponding to the question *does l-leucine treat cancer?*)
- An indication frame where *l-leucine* is the theme and *cancer* is a purpose (corresponding to *is l-leucine indicated for cancer?*)

Annotators were instructed to annotate a single frame in such cases and to take the question topic into consideration in making a decision. In this question, *l-leucine* is the more salient entity (thus, the question topic) and therefore, the frame in which this entity appears as the THEME element (i.e., the INDICATION frame) would be preferred. If both entities are equally likely as question topic, we asked the annotators to prefer specific question types over others (e.g., TREATMENT over INDICATION, COMPLICATION over EFFECT, CAUSE over DIAGNOSE_ME).

### Annotation confidence estimation

It became clear early on that adjudicating annotations in CHQA-email would be significantly difficult, due to the number and length of questions, and the size of annotator pool (21 annotator pairs). This is in contrast to CHQA-web with the smaller number of questions, which are shorter on average, and annotators (4 pairs). Thus, we investigated whether we can estimate confidences for the annotations.

For this purpose, we used two annotation confidence modeling methods: MACE (Multi-Annotator Competence Estimation) [67] and another probabilistic approach proposed by Passonneau and Carpenter (which we refer to as P&C here) [68]. Implementations of both methods are freely available and have been successfully applied to crowd-sourced data. They share similarities: they are both generative models based on variations of the item-response model [69] and learn annotator competence and correct labels in an unsupervised fashion using expectation-maximization (EM). They mainly differ in priors and the EM flavor used, model paramaters, and goals. We refer the reader to respective papers for further details.

We considered annotations of named entities, topic, triggers, and frames for modeling. To simplify matters, we treated each annotation as an independent data instance for MACE and P&C. Once we modeled the annotation confidences using these methods, we used the confidence scores generated to create a new set of annotations. When incorporating these scores, we assumed that if two annotators agreed on a specific annotation, that annotation was correct. For the rest of the annotations on which two annotators did not agree, we followed the following procedure:

- We calculate the mean average confidence score for each semantic class (i.e., entities, topic, triggers, frames).
- If the confidence score associated with an individual item is higher than the mean average for its class, it is added to the new set of annotations as long as it is consistent with the annotations already added. Consistency is defined as follows:
  - If the item under consideration is named entity or question trigger, it is always considered consistent, as they are atomic and independent.
  - If it is a question topic, it is considered consistent as long as the underlying named entity has already been added.
  - If it is a question frame, it is consistent as long as its trigger and all its arguments have already been added.

We compared these two methods to two other simple methods: the first (AGREE) only considers the subset of annotations that annotators agreed upon for the new set. The second (IAA_RANK) uses inter-annotator agreement results. For this method, we rank annotators by their inter-annotator agreement with others. Then, for a given question, we prefer the annotations of the annotator ranked higher among the two who annotated the question. Note that none of the methods as used here can distinguish cases where both annotators agree but are both incorrect.

To evaluate the performance of these methods, we ran experiments on CHQA-web, since an adjudicated set of annotations was available. Based on the results we obtained, we generated several sets of annotations for CHQA-email incorporating these methods. We make these sets publicly available in addition to raw annotations by each annotator.

## Results and discussion

In this section, we present and discuss corpus statistics for each part of the corpus, provide an in-depth analysis of the semantic categories annotated, detail inter-annotator agreement and annotation confidence estimation results.

### Corpus Statistics

Table 7 shows the basic corpus statistics for each part of the corpus. On average, CHQA-email requests are much longer (55.1 tokens vs. 7.5), confirming findings of earlier research [3, 8]. Standard deviation is high, reflecting a large amount of variation in question length.

**Table 7:** Basic corpus statistics

Corpus Part	# questions	# tokens	Average	Range	Std. Dev.
CHQA-email	1,740	95,834	55.1	2-427	51.3
*- Practice*	20				
*- Unadjudicated*	1,720				
CHQA-web	874	6,597	7.5	3-51	4.1
Total	2,614	102,431	39.2	2-427	136.7

### Named entity annotation

**Fig. 2:**
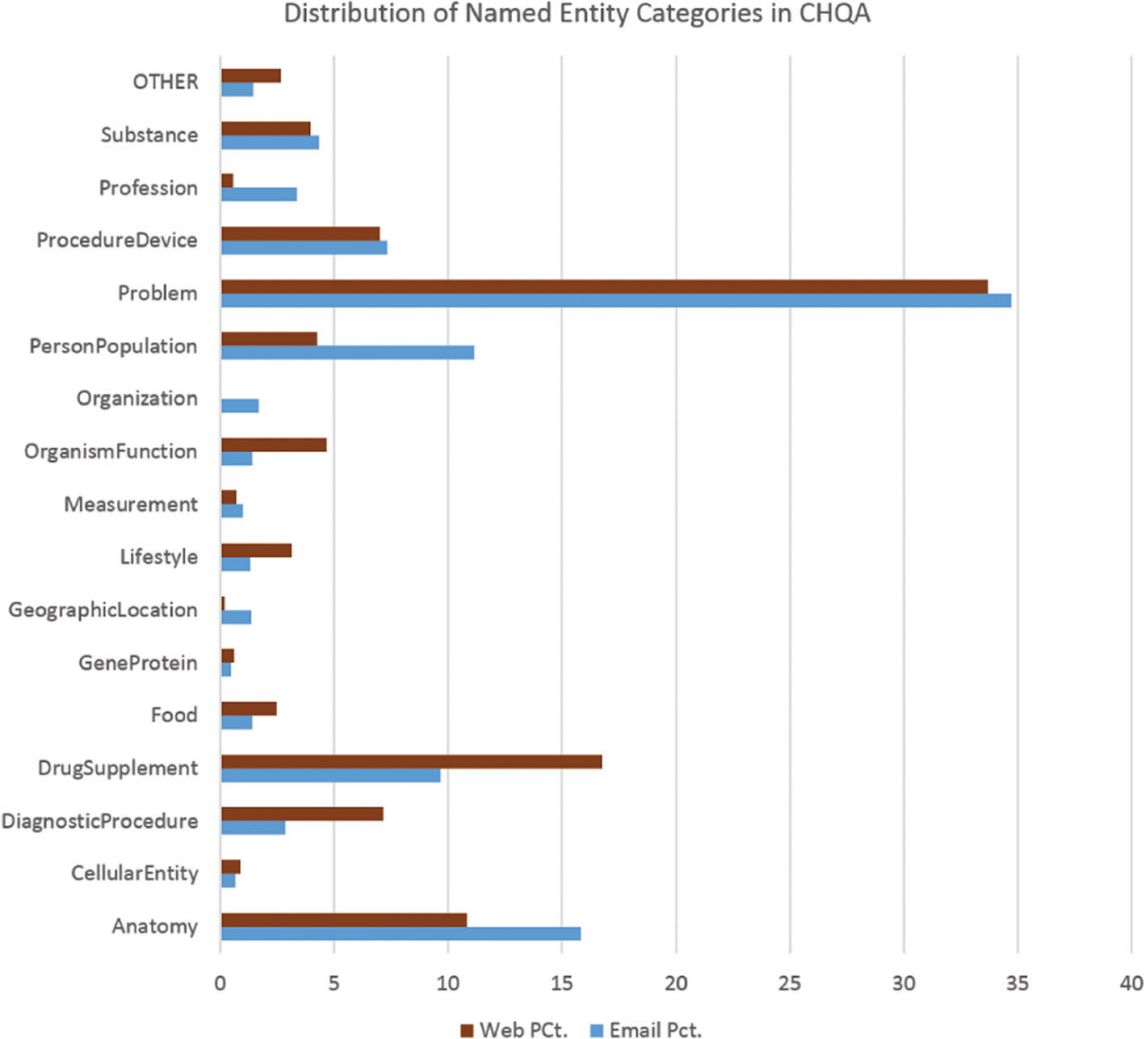
The distribution of named entities in CHQA-email and CHQA-web parts of the corpus. RESEARCH_CUE, annotated in only CHQA-web, is not included

Statistics regarding named entities in the corpus are provided in Table 8. Results for CHQA-email are presented separately for the small practice set and the larger unadjudicated set, because the questions in the latter have been double-annotated and each question is counted twice. The comparison of frequency of the named entity categories is also illustrated in Fig. 2.

**Table 8:** The distribution of annotated named entity categories

Category	# questions	% (Rank)	# questions	% (Rank)	# questions	% (Rank)
	*CHQA-email*				*CHQA-web*	
	*Practice*		*Unadjudicated*			
anatomy	31	12.8 (4)	5,339	15.8 (2)	153	10.8 (3)
cellular_ entity	0	0 (17)	224	0.7 (16)	13	0.9 (12)
diagnostic_ procedure	3	1.2 (8)	967	2.9 (8)	101	7.2 (4)
drug_supplement	26	10.8 (5)	3,264	9.7 (4)	237	16.8 (2)
food	3	1.2 (8)	474	1.4 (11)	35	2.5 (11)
gene_protein	1	0.4 (16)	156	0.5 (17)	9	0.6 (14)
geographic_ location	2	0.8 (13)	455	1.4 (13)	3	0.2 (17)
lifestyle	2	0.8 (13)	438	1.3 (14)	44	3.1 (9)
measurement	3	1.2 (8)	331	1.0 (15)	10	0.7 (13)
organism_ function	3	1.2 (8)	469	1.4 (12)	66	4.7 (6)
organization	7	2.9 (7)	576	1.7 (9)	1	0.1 (18)
person_ population	36	14.9 (2)	3,763	11.2 (3)	60	4.2 (7)
problem	75	31.0 (1)	11,711	34.7 (1)	476	33.7 (1)
procedure_ device	32	13.2 (3)	2,481	7.4 (5)	99	7.0 (5)
profession	2	0.8 (13)	1,144	3.4 (7)	8	0.6 (15)
research_cue	-	-	-	-	4	0.3 (16)
substance	13	5.4 (6)	1,466	4.3 (6)	56	4.0 (8)
other	3	1.2 (8)	489	1.5 (10)	38	2.7 (10)
Total	242	100.0	33,747	100.0	1,413	100.0
Average	12.1		9.8		1.6	
Range	1-35		1-84		1-5	

Note that questions in the unadjudicated set are counted twice since this set is double-annotated

As expected, the distribution of named entity categories and the average number of annotations per question in CHQA-email are similar to those reported for CHQ-NER, which CHQA-email supersedes. For the short questions in CHQA-web, the average number of entities per question is much lower (1.6). DRUG_SUPPLEMENT and DIAGNOSTIC_PROCEDURE terms appear more frequently in CHQA-web, while categories like PERSON_POPULATION, ORGANIZATION, PROFESSION, which often provide background information, appear less frequently than in CHQA-email. The distribution also confirms findings from earlier studies that consumer questions focus mostly on diseases and symptoms (i.e., PROBLEM category).

A small number of named entity annotations were added to CHQA-email (Unadjudicated) or deleted by annotators, increasing the number of annotations in this set from 33,494 to 33,747. PROBLEM category had the highest number of additions (141), and ORGANIZATION had the highest number of deletions (12), though additions/deletions often amounted to a very small percentage of the entities in a given category. Additions included problems like *pain in period* and drug classes like *allopathic medicines*.

RESEARCH_CUE category was only annotated in CHQA-web. In CHQA-email, RESEARCH attribute assigned to question frames indicates the same information without explicitly marking the mention. The total number of RESEARCH attribute annotations are 2 and 109 in CHQA-email (Practice) and CHQA-email (Unadjudicated), corresponding to 0.8% and 0.3% of all the entities, respectively, indicating a distribution similar to that of RESEARCH_CUE in CHQA-web.

### Question trigger/type annotation

**Fig. 3:**
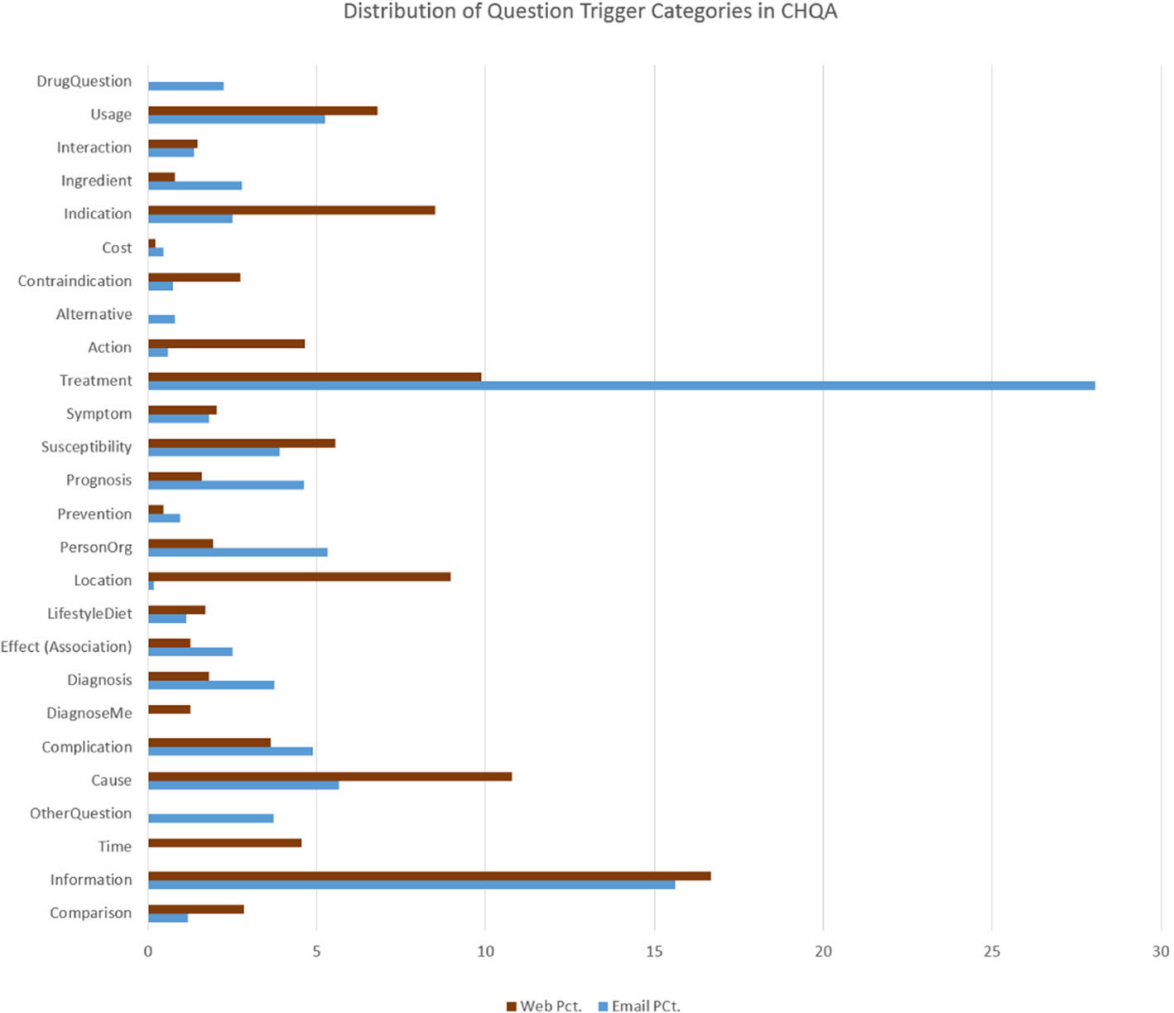
The distribution of question trigger types in CHQA-email and CHQA-web parts of the corpus. The question type categories in CHQA-web is used and some of the CHQA-email types are merged with their supertypes (e.g., SUPPORT_GROUP is merged with PERSON_ORGANIZATION) for simplicity

Statistics regarding question triggers/types in the corpus are provided in Table 9. TREATMENT triggers are most common in CHQA-email by a significant margin. In CHQA-web, INFORMATION triggers are most common, though its margin over the next most frequent type, CAUSE, is not as large. There are differences in the distribution of question types between the two parts: most significant are PERSON_ORGANIZATION and PROGNOSIS question types, which appear much more frequently in CHQA-email, and CAUSE, LOCATION, ACTION, and INDICATION question types, which occur more frequently in CHQA-web. Some of these differences can be explained by the question selection methodology. For example, in constructing CHQA-web, the same number of questions were sampled using different *wh*-words. *where* questions often contain LOCATION questions, which occurs in large quantity in CHQA-web, but is very rare in CHQA-email. The comparison of frequency of question trigger types in CHQA-email and CHQA-web is illustrated in Fig. 3. The distribution of question types in CHQA-email is similar to that presented for GARD dataset in Roberts et al. [10].

**Table 9:** The distribution of annotated question triggers/types

Category	# questions	# questions	% (Rank)	# questions	% (Rank)
	*CHQA-email*			*CHQA-web*	
	*Practice*	*Unadjudicated*			
*General question types*					
comparison	1	52	1.2 (19)	25	2.8 (11)
information	1	692	15.6 (2)	147	16.7 (1)
time	-	-	-	40	4.5 (9)
other_question	0	165	3.7 (7)	0	0 (24)
*Problem question types*					
cause	4	251	5.7 (3)	95	10.8 (2)
complication	1	66	1.5 (16)	32	3.6 (10)
diagnose_me	-	-	-	11	1.3 (19)
diagnosis	0	166	3.8 (6)	16	1.8 (15)
effect (association)	0	111	2.5 (10)	11	1.3 (19)
frequency	0	20	0.5 (29)	-	-
inheritance	0	87	2.0 (14)	-	-
lifestyle_diet	0	50	1.1 (20)	15	1.7 (16)
location	0	8	0.2 (32)	79	9.0 (4)
person_organization	1	224	5.1 (4)	17	1.9 (14)
prevention	0	42	1.0 (21)	4	0.5 (22)
prognosis	1	205	4.6 (5)	14	1.6 (17)
support_group	0	12	0.3 (31)	-	-
susceptibility	0	66	1.5 (16)	49	5.6 (7)
symptom	0	80	1.8 (15)	18	2.0 (13)
treatment	9	1,243	28.1 (1)	87	9.9 (3)
*Intervention question types*					
action	0	26	0.6 (28)	41	4.7 (8)
alternative	0	35	0.8 (22)	0	0 (24)
contraindication	1	33	0.7 (25)	24	2.7 (12)
cost	1	20	0.5 (29)	2	0.2 (23)
dosage	0	34	0.8 (23)	-	-
indication	1	111	2.5 (10)	75	8.5 (5)
ingredient	1	123	2.8 (9)	7	0.8 (21)
interaction	0	60	1.4 (18)	13	1.5 (18)
long_term_effect	1	33	0.7 (25)	-	-
overdose	1	8	0.2 (32)	-	-
side_effect	1	109	2.5 (12)	-	-
storage_disposal	0	31	0.7 (27)	-	-
tapering	0	34	0.8 (23)	-	-
usage	0	133	3.0 (8)	60	6.8 (6)
drug_question	0	99	2.2 (13)	0	0 (24)
Total	25	4,429		882	
Average	1.25	1.29		1.01	
Range	1-4	1-15		1-2	

Note that questions in the unadjudicated set are counted twice since this set is double-annotated

We analyzed the distribution of trigger expressions for each question type. A subset of results is provided in Table 10. Only top 10 triggers, if they occur more than once, are listed. We list the triggers without any lemmatization or spelling correction. Our analysis indicates that trigger lists for question types often have a long tail. In CHQA-email, TREATMENT, COST, and CAUSE question types have the least uniform distribution (i.e., relatively few triggers are used), while in CHQA-web, these question types are LOCATION and CAUSE. There is a slight preference for verbal triggers in CHQA-web as compared with CHQA-email. Light verbs, such as *get*, *help*, *have*, and *do* are the most heterogeneously annotated triggers, indicating 13, 11, 11, and 11 different question types, respectively, in CHQA-email. In CHQA-web, these triggers are *have*, *why*, *when*, and *take*, indicating a mix of light verbs and *wh*-words, to some extent reflecting the question selection process for this part of the corpus.

**Table 10:** The distribution of annotated question triggers

Category	#	Top triggers	#	Top triggers
	*CHQA-email (Unadjudicated)*		*CHQA-web*	
*General question types*				
comparison	28	*difference (10), better, differences, better then, vs, versus, similiar, like, comparison, compared*	20	*better (5), most effective*
information	272	*information (144), info, know, help, is, research, mean, learn*	52	*what is (56), is, normal, why, which is, called, what, means, mean, ?*
time	-	-	23	*after (8), when, stay, take, start, how long*
other_question	131	*danger (5), cost (5), too low, low, long*	0	-
*Problem question types*				
cause	70	*cause (76), causes, caused, causing, why, reason*	26	*why (31), cause, causes, when, is, ?*
complication	40	*cause (6), complications (6), risks, effect, caused, affect*	29	*result (2), after, affect*
diagnose_me	-	-	10	*problem (2)*
diagnosis	85	*test (13), diagnosis(13), tests, testing, tested, what, for, exams, detected*	11	*show (6)*
effect (association)	69	*effect(7), interfere, due to, connection, cause, related, link, affect*	8	*affect (3), do with*
frequency	14	*common (5), uncommon*	-	
inheritance	54	*genetic (9), passed, inherited, chance, carriers, passed down, pass*	-	
lifestyle_diet	37	*eat (5), food, maintain, is, help, exercises, do, diet, avoid*	11	*need (2), good, feed, drink*
location	7	*most common location (2)*	21	*where (23), where is, located, where are, go, come from*
person_ organization	114	*doctor (12), where, contact, find, specialist, place, study, go, doctors, anyone*	13	*who (3), performs, administer*
prevention	22	*prevent (12), protect, prevention, prevented, avoid, privention*	2	*prevent (3)*
prognosis	148	*how long (8), prognosis, go away, recovery, life expectancy, happen, lead to, continue*	13	*happen (2)*
support_group	10	*support (3)*	-	
susceptibility	41	*get (10), risk, contagious, expose, transmitted, start again, spread, passed, occur, go to the hospital*	29	*contagious (8), affected, gets, common, affect, risk*
symptom	51	*symptoms (24), signs, normal, symptom, neck symptoms, mouth symptoms, heart symptoms, get, feeling, do*	14	*know (3), symptoms, affect*
treatment	303	*treatment (202), help, do, cure, treatments, treat, medicine, stop, for, take*	46	*treat (9), help, used, lower, for, cure, treatment, stop, reduce, increase*
Category	#	Top triggers	#	Top triggers
	*CHQA-email (Unadjudicated)*		*CHQA-web*	
*Intervention question types*				
action	21	*how long (3), help, half life, effects*	29	*why (4), works, work, used, responsible, help, go, controls*
alternative	25	*alternative (6), take, superior, substitute, replacements, alternatives*	0	-
contraindication	23	*take (5), use, should not take, hurt, have*	22	*bad (2), after (2)*
cost	5	*cost (13), generic, prices*	1	*cost (2)*
dosage	19	*dosage (10), administration rates, lowest starting dose, dose, concentration*	0	-
indication	73	*help (10), for, used, take, prescribed, get*	34	*need (9), why, use, take, get, used, have, for*
ingredient	38	*is (26), contain, ingredients, in, free, contains, chemicals, ingredient, are*	4	*contain (3), is*
interaction	41	*together (5), take, safe*	12	*when taking (2)*
long_term_effect	26	*safe (2), long term effects, long term adverse reactions, last, damage, contributed, cause*	0	-
overdose	8	-	0	-
side_effect	60	*side effects (16), cause, side affect, side effect, effect, have, effects, causes, affect*	0	-
storage_disposal	21	*storage (3), stability (3), mixing (3), storing, kept, keep, glycogen storage disease, effective*	0	-
tapering	28	*cut back (2), orwithdraw, tapering, withdrawal, weaning, wean*	0	-
usage	89	*use (8), take, taken, taking, get, instructions, how long, doing*	42	*use (12), stop, given, take, dosage*

Numbers in the second and fourth columns are unique counts of triggers used for the corresponding category. Only triggers that occur at least twice are shown. The most frequent trigger for a given category is indicated with its frequency in parentheses (when this frequency is 2, all triggers given occur twice)

We asked annotators to indicate their proposed question types when annotating OTHER_QUESTION and DRUG_QUESTION categories in CHQA-email. After the annotations were completed, we manually categorized the small number of proposed types the annotators had specified. These types are provided in Table 11. Reference ranges for diagnostic tests, availability of drugs and other interventions as well as DIAGNOSE_ME (which we adopted later for CHQA-web) were among the most frequent. In general, the annotators were not consistent in proposing new question types; therefore, these results should be interpreted with caution. Also note that the annotation guidelines sometimes indicate a particular question type for a specific kind of information request annotated with one of these two types (e.g., INFORMATION for Manufacturer); however, these were missed by some annotators, indicating potential annotation errors.

**Table 11:** The distribution of proposed question types annotated as OTHER_QUESTION or DRUG_QUESTION

Category	# questions	Brief desription
other_question		
Antidote	2	How to deal with a chemical
Availability	9	Availability of an intervention on the market, where to get it
Complication management	1	How to fix an issue arising from a procedure
Contraindicated	1	What is contraindicated for a disease
Diagnose Me	5	Diagnosis given a list of symptoms
Duration	3	How long for a procedure/treatment
Fertility	1	Possible to have children with existing condition
Function	1	How a body part works
Gene-disease association	2	Association between a gene and a disease
History	3	History of a disease
Incubation	1	Incubation period for a disease
Interpretation	5	Lab result interpretation
Post-procedure management	3	Management options after a procedure
Preparation	2	How to prepare for a lab test
Procedure follow-up	7	Whether procedures are still needed after a problem is solved
Progression	3	How a disease progresses
Test result range	10	Reference values for a lab test/procedure
drug_question		
Clinical trial	3	Trials for a drug
Coindication	1	Whether to use a drug with another
Coverage	5	Whether insurance pays for a drug
Effect duration	3	How long the effect lasts
Form	4	What form the drug comes in
Manufacturer	6	Manufacturer of a drug
Packaging	1	How a drug is packaged
Pharmacokinetics	6	How long it takes for a drug to have effect
Potency	3	Whether a drug retains its potency after a time period
Prescription	4	Whether a prescription is needed
Stability	1	Whether a drug is stable when diluted
Transmission	3	Whether a drug is transmitted through body fluids

### Question frame annotation

**Fig. 4:**
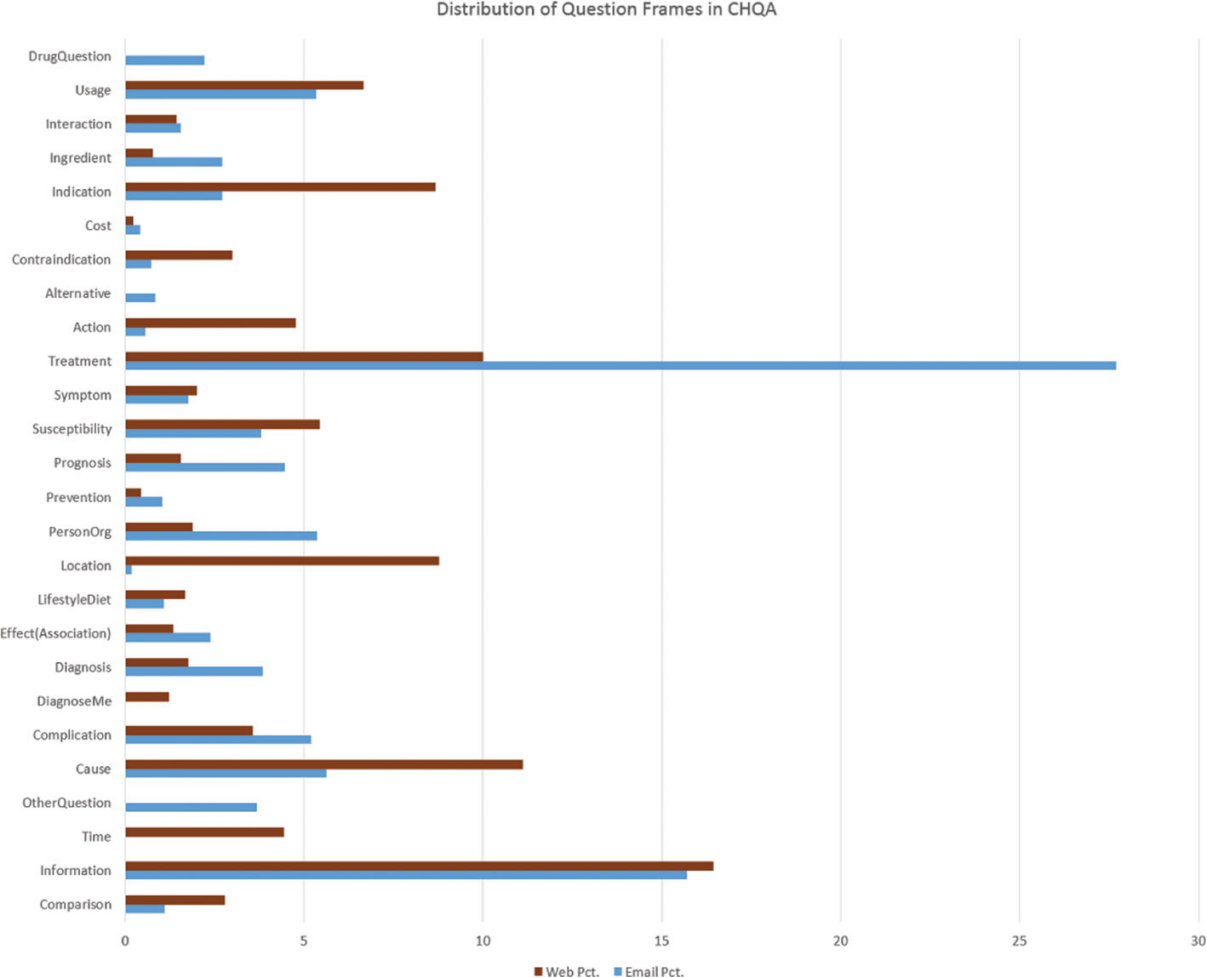
The distribution of question frames in CHQA-email and CHQA-web parts of the corpus. The question frame categories in CHQA-web is used and some of the CHQA-email types are merged with their supertypes

Statistics regarding question frame types in the corpus are provided in Table 12. For CHQA-email (Practice), the results are the same as those for question triggers, indicating that each trigger in this set was associated with a single frame. In CHQA-email (Unadjudicated) and CHQA-web, a small proportion of triggers were used by several frames. The maximum number of frames indicated by a single trigger was 6. On average, a single SIDE_EFFECT trigger was annotated with highest number of frames, indicating that consumers were often interested in side effects of multiple drugs in the same question. The comparison of frequency of question frame categories in CHQA-email and CHQA-web is illustrated in Fig 4.

**Table 12:** The distribution of annotated question frame categories

Category	# questions	# questions	% (Rank)	# questions	% (Rank)
	*CHQA-email*			*CHQA-web*	
	*Practice*	*Unadjudicated*			
*General question types*					
comparison	1	52	1.1 (19)	25	2.7 (12)
information	1	736	15.7 (2)	148	16.5 (1)
time	-	-	-	40	4.5 (9)
other_question	0	172	3.7 (7)	0	0 (24)
*Problem question types*					
cause	4	263	5.6 (3)	100	11.1 (2)
complication	1	67	1.4 (17)	32	3.6 (10)
diagnose_me	-	-	-	11	1.2 (20)
diagnosis	0	180	3.8 (6)	16	1.8 (15)
effect (association)	0	112	2.4 (12)	12	1.3 (19)
frequency	0	21	0.5 (29)	-	-
inheritance	0	90	1.9 (14)	-	-
lifestyle_diet	0	51	1.1 (20)	15	1.7 (16)
location	0	8	0.2 (32)	79	8.8 (4)
person_organization	1	237	5.1 (4)	17	1.9 (14)
prevention	0	48	1.0 (21)	4	0.5 (22)
prognosis	1	209	4.5 (5)	14	1.6 (17)
support_group	0	14	0.3 (31)	-	-
susceptibility	0	67	1.4 (17)	49	5.5 (7)
symptom	0	83	1.8 (15)	18	2.0 (13)
treatment	9	1,298	27.7 (1)	90	10.0 (3)
*Intervention question types*					
action	0	26	0.6 (28)	43	4.8 (8)
alternative	0	39	0.8 (22)	0	0 (24)
contraindication	1	34	0.7 (26)	27	3.0 (11)
cost	1	20	0.4 (30)	2	0.2 (23)
dosage	0	36	0.8 (23)	-	-
indication	1	127	2.7 (10)	78	8.7 (5)
ingredient	1	127	2.7 (10)	7	0.8 (21)
interaction	0	73	1.6 (16)	13	1.5 (18)
long_term_effect	1	34	0.7 (26)	-	-
overdose	1	8	0.2 (32)	-	-
side_effect	1	134	2.9 (9)	-	-
storage_disposal	0	36	0.8 (23)	-	-
tapering	0	35	0.7 (25)	-	-
usage	0	143	3.1 (8)	60	6.7 (6)
drug_question	0	104	2.2 (13)	0	0 (24)
Total	25	4,684		900	
Range	1-4	1-18		1-2	
Average	1.25	1.36		1.03	

Table 13 shows the counts of semantic roles in frames. The percentage of frame annotations involving a non-THEME role was approximately 68% in CHQA-email and 46% in CHQA-web. This difference is to be expected, as longer CHQA-email questions often provide more context that may be relevant for answering questions. The most frequently used semantic role in CHQA-web was the unspecific RESTRICTOR. Considering that this role is largely similar to KEYWORD and not very informative, fine-grained annotation of semantic roles (AGENT, PATIENT, etc.) may not provide much advantage over simply using coarse-grained KEYWORD when it comes to question understanding. EXCLUDE_KEYWORD role was rarely used in CHQA-email, suggesting that its utility for answering consumer health questions may be limited, and was later dropped for CHQA-web.

**Table 13:** The distribution of frame semantic roles

Category	# questions	Range	# questions	Range	# questions	Range
	*CHQA-email*				*CHQA-web*	
	*Practice*		*Unadjudicated*			
theme	27	1-3	4,860	1-7	958	1-5
keyword	18	1-2	2,959	0-9	-	-
exclude_keyword	0	-	107	0-6	-	-
agent	-	-	-	-	91	0-2
locative	-	-	-	-	85	0-2
patient	-	-	-	-	58	0-1
purpose	-	-	-	-	24	0-1
restrictor	-	-	-	-	185	0-3
temporal	-	-	-	-	28	0-1
research	-	-	-	-	4	0-1

### Question topic annotation

We only annotated question topic in CHQA-email, as this element almost always corresponds to the theme element of the question in the short CHQA-web questions. On average, 1.8 question topics were annotated per question. The maximum number of topic mentions annotated for a single question was 17.

### Inter-annotator agreement

We calculated pairwise inter-annotator agreement, using micro-average F_1_ score when one set of annotations is taken as the gold standard. Agreement was calculated for named entities, question triggers/types, question topics, and frames. Exact span match criterion was used to compare mentions (named entities and question triggers).

We calculated frame agreement in several ways. For *full frame* agreement calculation, we considered the question trigger/type and all semantic roles (KEYWORD, AGENT, etc.). For *core frame* agreement, we considered only the THEME role in addition to question trigger/type. In addition, frame agreement calculation can be based on question trigger mentions or normalized question types indicated by these mentions. Combinations of these options, consequently, led to the following frame agreement types: *Full frame w/ trigger*, *Full frame w/ type*, *Core frame w/ trigger*, and *Core frame w/ type*. We consider *Core frame w/ type* as the most relevant for QA, as identifying specific words used to express a question precisely is generally not needed for accurately answering it.

**Fig. 5:**
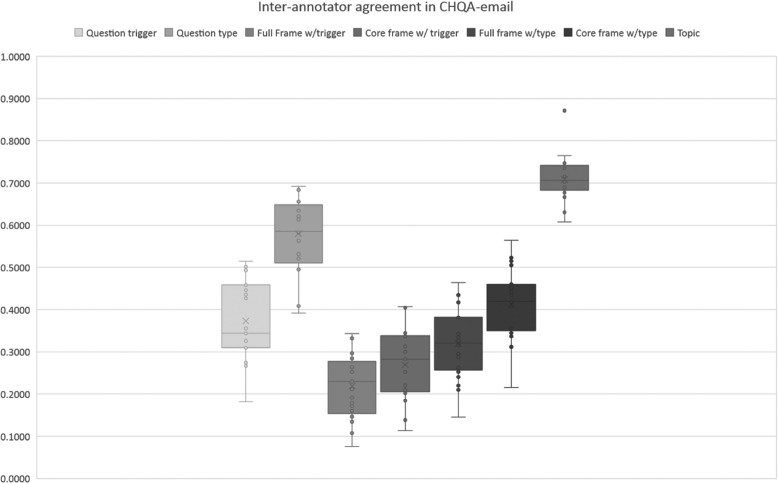
Inter-annotator agreement for various question elements in CHQA-email. Exact match criterion is used as the basis of agreement

**Fig. 6:**
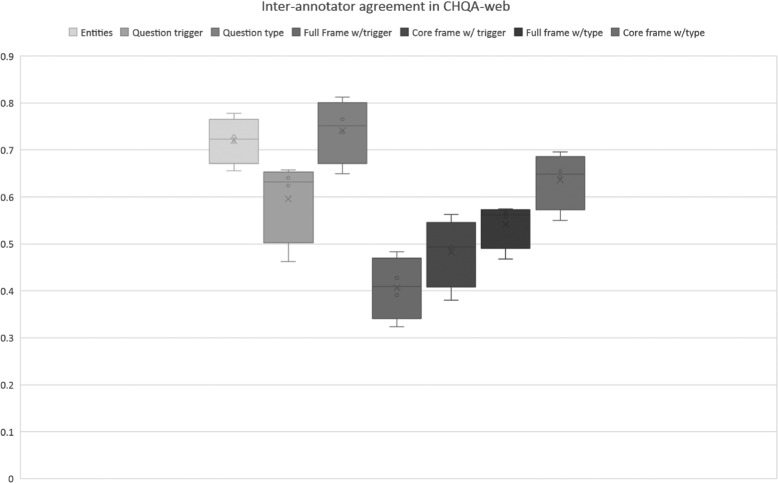
Inter-annotator agreement for various question elements in CHQA-web. Exact match criterion is used as the basis of agreement

We did not consider agreement on practice sets for each part of the corpus. We also did not calculate named entity agreement on CHQA-email (Unadjudicated), since this largely overlaps with CHQ-NER and the inter-annotator agreement for named entities in that set were reported earlier [12]. The results of inter-annotator agreement calculation are provided in Table 14 and illustrated in Figs. 5 and 6.

**Table 14:** Inter-annotator agreement results

Category	Average	Range	Average	Range
	*CHQA-email*		*CHQA-web*	
	*Unadjudicated*			
Avg. # of questions shared	81.9	36-120	540	540-540
Named entity	-	-	0.72	0.66-0.78
Question trigger	0.37	0.18-0.52	0.60	0.46-0.66
Question type	0.58	0.39-0.69	0.74	0.65-0.81
Full frame w/ trigger	0.22	0.08-0.34	0.41	0.33-0.48
Core frame w/ trigger	0.27	0.11-0.41	0.48	0.38-0.56
Full frame w/ type	0.32	0.15-0.46	0.54	0.47-0.58
Core frame w/ type	0.41	0.22-0.56	0.64	0.55-0.70
Question topic	0.71	0.61-0.87	-	-

Inter-annotator agreement is calculated as the micro-average F1 score when one set of annotations is taken as the gold standard

Inter-annotator agreement in CHQA-email is overall low. The highest agreement is for question topics (0.71) and the lowest is for full frame agreement (0.22), which considers named entity and trigger mentions, and all semantic roles (*Full frame w/ trigger*). Frame annotation in general is challenging as the results indicate, but the agreement is somewhat improved when the agreement only focuses on core frame elements, question type and theme (0.41 with *Core frame w/ type*). The low agreement for frames is not unexpected, as they can involve many parts, each potentially leading to disagreement. In particular, annotators had difficulty in agreeing on the exact boundaries of trigger mentions (0.37), one of the major components of the frames. When the matching criterion is changed to allow mention overlap (approximate match), average question trigger agreement increases from 0.37 to 0.45, full frame agreement from 0.22 to 0.28, and core frame with type agreement from 0.41 to 0.44. While these figures are still low, they suggest that more strict guidelines may be useful in annotating triggers. In contrast to named entities which are often expressed with specific part-of-speech elements, triggers are lexically and syntactically more heterogeneous and ambiguous, especially in consumer health questions; thus, developing such guidelines may be difficult. Analyzing inter-annotator agreement for triggers of each question type, we find that the agreement is generally higher for triggers of the frequent question types (Table 15). The question types ranked highest in terms of inter-annotator agreement for triggers are CAUSE (0.60), TREATMENT (0.52), PREVENTION (0.44), and PERSON_ORGANIZATION (0.43), while for the rare types, the agreement is very low or even zero (for SUPPORT_GROUP and OVERDOSE).

**Table 15:** Inter-annotator agreement broken down by question types and corresponding triggers

Category	Trigger	Type	Trigger	Type
	*CHQA-email*		*CHQA-web*	
*General question types*				
comparison	0.28	0.43	0.66	0.77
information	0.37	0.51	0.65	0.75
time	-	-	0.47	0.65
other_question	0.23	0.39	0.00	0.00
*Problem question types*				
cause	0.60	0.64	0.63	0.76
complication	0.17	0.21	0.58	0.64
diagnose_me	-	-	0.72	0.82
diagnosis	0.30	0.48	0.51	0.72
effect (association)	0.35	0.45	0.17	0.17
frequency	0.20	0.30	-	-
inheritance	0.30	0.46	-	-
lifestyle_diet	0.02	0.25	0.32	0.37
location	0.20	0.20	0.60	0.85
person_organization	0.43	0.63	0.18	0.63
prevention	0.44	0.51	0.50	0.50
prognosis	0.25	0.49	0.41	0.77
support_group	0.00	0.00	-	-
susceptibility	0.26	0.34	0.57	0.73
symptom	0.26	0.45	0.53	0.65
treatment	0.52	0.75	0.76	0.86
*Intervention question types*				
action	0.03	0.19	0.57	0.60
alternative	0.18	0.22	-	-
contraindication	0.10	0.25	0.57	0.67
cost	0.40	0.40	1.00	1.00
dosage	0.23	0.37	-	-
indication	0.15	0.29	0.48	0.75
ingredient	0.36	0.83	0.50	0.50
interaction	0.20	0.73	0.57	0.78
long_term_effect	0.22	0.30	-	-
overdose	0.00	0.33	-	-
side_effect	0.31	0.39	-	-
storage_disposal	0.19	0.35	-	-
tapering	0.13	0.36	-	-
usage	0.08	0.40	0.62	0.72
drug_question	0.14	0.28	-	-

Compared to question triggers, the agreement on question types is much higher, indicating that annotators can identify the information request in abstract, but have difficulty in annotating the relevant piece of text. We also note that agreement for question types is lower than that reported for GARD (0.58 and 0.74 in this study vs. 0.81) [10]. This is partly due to the fact that the number of question types is significantly higher in our case and also to the fact that GARD questions are in general much shorter and well-formed. On the whole, these results point out that it would be more practical to focus on annotating question types only, instead of attempting to also precisely identify the question triggers. However, we should also note that machine learning approaches are likely to benefit from mention-level trigger annotations.

Agreement in CHQA-web is consistently higher than that in CHQA-email by about 0.2 points. Trends are similar for different semantic classes, confirming our basic intuition that annotating these shorter questions is easier, compared to CHQA-email. Agreement for named entities (0.72) is consistent with that reported for CHQ-NER (0.71) [12], although given the trends, we would have expected it to be higher. This may be attributed to the fact named entity annotation for this part of the corpus was not performed as a separate study, and annotators may not have been as diligent in following the strict guidelines for named entity annotation. In this part of the corpus, inter-annotator agreement for question triggers is generally higher than that in CHQA-email, and is more evenly distributed between frequent and rare question types: among the top four question types are COST, DIAGNOSE_ME, and COMPARISON, which are relatively rare, as well as TREATMENT, the third most frequent trigger type in this subset (Table 15). Agreement on question types follows a similar pattern and, for frequent question types, reaches as high as 0.86 (TREATMENT).

### Annotation confidence estimation

The evaluation results of annotation confidence estimation for CHQA-web are provided in Table 16. These results have been obtained by comparing the estimation method results with the adjudicated results. We ran the same estimation methods on CHQA-email (Unadjudicated) and provide the resulting data; however, we were unable to evaluate those results, as no gold standard exists for comparison.

**Table 16:** Annotation confidence estimation in CHQA-web

Method	Precision	Recall	F_1_
AGREE	0.99	0.66	0.79
IAA_RANK (*Full frame w/ trigger*)	0.78	0.80	0.79
IAA_RANK (*Core frame w/ type*)	0.85	0.84	0.84
MACE	0.82	0.86	0.84
MACE (Reliability Rank)	0.83	0.83	0.83
P&C	0.87	0.79	0.83
P&C (Reliability Rank)	0.81	0.82	0.82

The results show that the baseline (AGREE: taking simple agreement of annotators as indication of confidence) already achieves an agreement of 0.79 with the adjudicated results. The precision of 0.99 indicates that a small number of annotations that the annotators agreed upon were discarded later in adjudication. All other methods improve on the low recall of the baseline (0.66), while leading to some precision loss. IAA_RANK uses the ranking of annotators by their average agreement score, and can be based on any of the agreement categories described earlier. Because frame annotations incorporate other atomic annotations (named entities, triggers), we ran IAA_RANK with two frame agreement results. The first ranks the annotators by their average agreement for full frame with triggers, and the second by their agreement for core frame with types. The latter led to best confidence estimation for CHQA-web (0.844).

The results obtained with MACE [67] and P&C [68] methods were similar to each other, although MACE had a slight edge and provided confidence estimation that is very close to the best results (0.841 vs. 0.844). Using the annotator reliability ranking provided by MACE and P&C, we obtained results that are slightly lower than their confidence score-based counterparts (0.83 vs. 0.84 and 0.82 vs. 0.83). Overall, the results obtained by these two methods are lower than the results reported for other corpora [67, 68]. We attribute this partly to the fact that crowdsourcing is used in those cases; with a larger pool of annotators who annotate all instances, it is likely that these methods would perform better. Furthermore, compared to the type of annotations they consider (e.g., word senses, textual entailment pairs), our annotations (in particular, frames) are significantly more complex. In fact, when we only focused on the named entities in CHQ-NER (1548 questions) (i.e., less complex annotations), we obtained the best results with MACE. As noted earlier, as input to these two methods, we made the simplifying assumption that each annotation is independent. A representation that better reflects the compositional nature of frames may also lead to better results.

We ran the same methods on CHQA-email (Unadjudicated) and provide this part of the corpus in several versions: AGREE, IAA_RANK (both *Full frame w/trigger* and *Core frame w/ type*), MACE, and P&C subsets. In addition, we provide MACE and P&C confidence estimations for each annotation. This can enable further research in developing more advanced methods that estimate annotation confidence. For example, the confidence scores generated by MACE and P&C and the output of other simpler methods can be used as features to learn to predict confidence. Confidence scores can also be used in an active learning framework, where the annotator is presented only with the annotations with moderate confidence, reducing adjudication load. Distinguishing high from low confidence items can also benefit training and evaluation: for example, a system can be penalized less for an incorrect response, when the annotation is labeled with low confidence [68].

## Conclusion

We presented a corpus of consumer health questions annotated for various semantic classes: named entities, question triggers/types, question topics, and frames. The questions cover a wide spectrum, from short questions with little to no context to long questions filled with irrelevant information, reflecting all the complexity and difficulty of consumer health questions. With respect to question understanding and answering, they present different challenges and different approaches may be needed. The corpus is the first focusing on annotation of real consumer questions, and with its size, the number and types of annotations included, it represents a rich resource for consumer health QA. The corpus, annotation guidelines and various analyses are publicly available at https://bionlp.nlm.nih.gov/CHIQAcollections/CHQA-Corpus-1.0.zip.

Our corpus has some limitations. Our conceptualization of question understanding and our goals have evolved over time and the corpus reflects this to some extent. Two parts of the corpus are largely similar, but there are also differences. In most cases, these differences can be reconciled with some automatic processing, as in question types that are merged in the other part of the corpus or non-THEME semantic roles. Some aspects are more difficult to reconcile, however. For example, we ignored diagnosis-seeking questions (DIAGNOSE_ME) for a long time, since they were considered outside the scope of our work. More recently, we began considering these questions but annotated them only in CHQA-web. A large portion of the corpus has not been adjudicated due to personnel/time constraints, which may limit its usefulness. To address this, we experimented with several methods of annotation confidence estimation and make these estimates available, which could stimulate research in better understanding annotator behavior and automatic adjudication.

In ongoing and future work, we plan to expand this corpus on several other semantic dimensions. For example, we are currently normalizing named entities in our corpus by mapping them to the corresponding UMLS Metathesaurus concepts. By addressing the lexical variability in named entities, this can improve question understanding. In earlier work, we found that coreference played a major role in consumer health question understanding [9], and we plan to add coreference annotations to a subset of CHQA-email. Similarly, we annotated spelling errors on a smaller set of long questions in an earlier study [58], and we also plan to add such annotations to this corpus.

## Footnotes

1
http://www.mdedge.com/jfponline/clinical-inquiries

2
https://rarediseases.info.nih.gov/

3 In previous work, we used the terms *focus* [11] and *topic* [57] to refer to this element. In this paper, we use the term *topic*.

4 Note, however, that named entities are reconciled since they have been mostly inherited from CHQ-NER [12].
